# Supplementary material for: Patterns of Intron Gain and Loss in Fungi
Source: PLoS Biol. 2004 Nov 30;2(12):e422. doi: 10.1371/journal.pbio.0020422 (PMC532390; doi:10.1371/journal.pbio.0020422)
Supplement: Table S1 — Also available at http://genes.mit.edu/NielsenEtAl/. (4.3 MB ZIP). [file pbio.0020422.st001.zip › NielsenEtAl/html/1386.html]

AN6655.1.NCU02480.1.MG02612.1.FG00177.1


```
 CLUSTAL W (1.82) Multiple Sequence Alignments - Introns Inserted


Sequence 1: AN6655.1	279 aa
Sequence 2: FG00177.1	283 aa
Sequence 3: MG02612.1	280 aa
Sequence 4: NCU02480.1	282 aa
Alignment Length: 285 aa
Number Identitical Residues: 196 aa
Alignment Score (without introns) 8460


MG02612.1 	---MAGRLAGKNAVITGAAG2GIGLETSILFAKEGAHVLMADISLDALAKALDKVKSLVP
NCU02480.1	---MPGRLAGKNAIITGAAG2GIGLETSILFAKEGANVLMADISAEAIQRAEAKVKQLVP
FG00177.1 	MSAPRGRLAGKNAVITGAGG2GIGLETSILFAKEGASILMSDISQPALEKAAAKVKQLVP
AN6655.1  	MATPRGRLQGKNAIITGAAG2GIGLETSILFAREGANVLMADISASALEKALAKVRELVP
          	 ::  *** ****:****.* ***********:*** :**:***  *: :*  **:.***

MG02612.1 	GA-QVETV0KCDVSKEAEVKAMIEHVDAWGGLDIIFNNAGIMHADDADAVDTPENIWNLT
NCU02480.1	NASRVESI0VCDVSKEDQVEKMVSHLDSWGGLDIIFNNAGIMHSQDDDAVATPESIWDLT
FG00177.1 	DAPRVEIL0KVDVSKESEVQAMIESLDSWGGIDVLFNNAGIMHAQDDDAVNTPENIWDLT
AN6655.1  	DAPRVETI~KCDVSKESEVQAMVESQDSWGGTDVIFNNAGIMHADDADAIDTPEKIWDLT
          	.*.:** :   ***** :*: *:.  *:*** *::********::* **: ***.**:**

MG02612.1 	QDINVKGVWYGSKHAVLALRRHKKTKGSIINTASVVALVGSATPQLAYTASKGAVLAMTR
NCU02480.1	HNINVKGVWFGCKHAVLSLRKHKKTKGSIINTASVVALVGSATPQLAYTASKGAVLAMTR
FG00177.1 	QNINVKGVWFGCKHAVLSLRRNKKSKASIINTASVVALVGAATPQLAYTASKGAVLALTR
AN6655.1  	QNINVKGVWFGCKHAVLSMRRHKKSKGSIINTASVVALVGSATPQLAYTASKGAVLALTR
          	::*******:*.*****::*::**:*.*************:****************:**

MG02612.1 	ELAIVHAREGFRFNNLCPAPLN~TPLLQDWLGDDQAKRHRREVHFPTGRFGEAVEQAQAV
NCU02480.1	ELAMVHAREGFRFNSLCPAPLN~TPLLQDWLGDDKAKRLRREIHFPTGRFGEAVEQAQAV
FG00177.1 	ELAMVHAREGFRFNNLCPAPLN~TPLLQDWLGDDQAKRHRREVHFPMGRFGEAIEQAHAV
AN6655.1  	ELAIVHAREGIRFNALCPAPLN2TPLLQDWLGDDQAKRHRREVHFPMGRFGEAIEQAHAV
          	***:******:*** ******* ***********:*** ***:*** ******:***:**

MG02612.1 	LFLASDESSFVNGHDFVVDGGMTK~AYVTPEGPAMAAPQNNASKSSLA-
NCU02480.1	VFLASDEASFVNGHDMVVDGGMTK~AYVTPEGPATAAPVNNALKESLEE
FG00177.1 	VFLASDESSFVNGHDFAVDGGMTK~GYVTAEGAAPPPPQNNASKDSL--
AN6655.1  	VFLASDESSFVNGADFVVDGGMSK0AYVTPEGPAPPAPKNQAQ------
          	:******:***** *:.*****:* .***.**.* ..* *:*
```
